# Risk of readmission following admission with community-acquired sepsis to a medical emergency department - a follow up study

**DOI:** 10.1186/1757-7241-23-S1-A28

**Published:** 2015-07-16

**Authors:** Daniel P Henriksen, Christian B Laursen, Annmarie T Lassen

**Affiliations:** 1 Medical Emergency Department, OUH Odense University Hospital, Odense, Denmark; 2 Department of Respiratory Medicine, OUH Odense University Hospital, Odense, Denmark

## Background

Sepsis is a common cause of admission to the hospital, and an increasing proportion of patients survive to discharge. Knowledge regarding risk of readmission might be of value in planning rehabilitation and follow up of these patients.

## Methods

We included all adult patients (≥15 years) with a first-time admission of community-acquired sepsis of any severity at a medical Emergency Department (ED) between September 2010 and August 2011 and who survived to discharge. Cases were identified by manual chart review using predefined criteria of infection. Data on vital signs, laboratory values, and antibiotic treatment were obtained electronically. We computed the median time to readmission and combined the severe sepsis and septic shock category due to the low number of patients with septic shock (N = 14). We used the Wilcoxon Rank-Sum test to test whether there was any difference in the median time to readmission among patients discharged after an admission with sepsis compared to severe sepsis/septic shock. Patients were followed until death, emigration, readmission to the hospital, or 90 days after discharge with sepsis of any severity, whichever came first.

## Results

A total of 1,713 patients were admitted with sepsis of any severity, and 1,542 (90.0%) were discharged. Within the first 90 days after discharge, 520 (33.7%) patients were readmitted, 84 (5.5%) died before being readmitted to the hospital, and 2 (0.1%) emigrated. A total of 178/600 (29.7%) patients discharged after an admission with sepsis were readmitted within 90-days after discharge and 342/942 (36.3%) were readmitted after severe sepsis/septic shock (p = 0.007). The median time of readmission was 21 days (IQR 8-44 days) among patients discharged after a hospitalization with sepsis and 20 days (IQR 8-41 days) in severe sepsis/septic shock (p = 0.691).

## Conclusion

One third of patients who survived an admission with sepsis of any severity were readmitted to the hospital within 90 days after discharge, and patients with severe sepsis/septic shock were more likely to be readmitted than patients with sepsis. There was no difference in the time to readmission when comparing patients discharged after sepsis and severe sepsis/septic shock.

